# Lectures on New Remedies and Their Therapeutical Applications

**Published:** 1862-05

**Authors:** Samuel R. Percy

**Affiliations:** Professor of Materia Medica and Therapeutics


### Selections.

### LECTURES ON NEW REMEDIES AND THEIR THERAPEUTICAL APPLICATIONS.

DELIVERED AT THE
NEW YORK MEDICAL COLLEGE AND CHARITY HOSPITAL.

By SAMUEL R. PERCY, M.D.,
*Professor of Materia Medica and Therapeutics.*

Reprinted from the American Medical Times.

### THE ACTIVE PRINCIPLE OF COLCHICUM—COLCHICINA.

With the principle which I have prepared, and which is identical with the *colchiceine* of Oberlin (and of the chemical differences between this and the *colchicin* of Geiger and Hasse I have already spoken,) I have tried some physiological experiments. To a full grown dog, weighing about twelve pounds, I administered one grain finely rubbed up with one drachm of sugar, and enveloped in a thin slice of meat; this was thrust down the throat. For about one hour no change was noticed, excepting a gradual increase in the frequency of the pulse, being at the end of the hour fifteen beats more than at the commencement. Gradually the dog began to show restlessness and pain. In two hours he had a full copious discharge from the bowels, the first portions of which were natural in appearance, but the latter portion was light colored, pultaceous, and very frothy; vomiting also commenced, which at first consisted of thin mucus but as the retching continued, the mucus thrown up was small in quantity, and freely tinged with blood. Urine was passed at first freely, and as an old dog usually passes it, but as the dog grew weaker many ineffectual attempts were made, and constant straining in the way which a young dog usually uses, without throwing up the leg; although the efforts to urinate were frequent, no urine was passed after three and a half hours. The pulse in two and a half hours was thin, wiry, and frequent. In six hours the pulse was small, feeble and reduced to twenty-four beats in the minute. In the meantime the diarrhœa had been very troublesome: the discharges were thin ochre-colored, frothy, with frequent patches of bloody mucus. After the seventh hour the dog did not attempt to rise, the pulse became small, thready, and intermittent, and he died a little before the eighth hour, without convulsions. Upon post mortem examination the heart contained much thick, pitchy-black blood, and the same also was found in the ascending and descending aorta as described by Bley, and even in the arteries of the legs and neck; the mucous membrane of the stomach was only slightly congested, but the whole mucous membrane of the small and large intestines was inflamed, even down to the anus, near which there were several large abrasions. Upon removing the kidney, and dividing it with a sharp knife, the first appearance was one reddened inflamed mass, and upon more minute examination the Malpighian bodies were very red and much congested, the interlobular plexus was also very much congested, and the congestion extended even to the infundibula and pelvis of the kidney.

Two grains were given to another dog, which died in eleven and a half hours, with all the symptoms above described. Towards the pyloric extremity of the stomach there was an irregular patch of about the size of a dollar, highly congested; the other parts of the mucous membrane were not much changed; the small and large intestines were like those in the other dog; the heart contained the same black, pitch-like blood; the kidneys were if anything more congested than those before described; the bladder was entirely empty.

To a dog, weighing about fourteen pounds, two grains of this colchicina were given finely rubbed up with a drachm of sugar, and a scruple of tannic acid. The whole was enveloped in a slice of meat, and pushed down the throat. The restlessness of the dog seemed greater than with either of the others described. There was retching in half an hour, and in about an hour free vomiting. In an hour and a half there was copious diarrhœa. Thirty grains of tannic acid were given, but it did not control the diarrhœa; there was not the same desire to urinate as shown by the other dogs, but no urine was passed after the fourth hour. The dog died in fourteen hours. The post mortem appearances were nearly the same as in those described, excepting that the kidneys were not so generally inflamed, though there was great congestion of the Malpighian bodies and interlobular plexuses, but it did not extend beyond them as in the other case; the bladder contained about a teaspoonful of very dark-colored urine. Thus tannic acid is no antidote to colchicina.

To a cat, while under the influence of chloroform, was administered one grain of colchicina, enveloped in a small round ball of bread. In little more than an hour it purged her freely, and produced very great uneasiness, as she prowled round in a restless and timid way, and constantly moaned. The tenesmus with the dogs was great, but with this cat it was very severe, so long as the strength lasted she seemed to make almost one continual strain. The cat died in eight hours. There was congestion of the stomach, intestines, and lungs; the right side of the heart was empty, but the left ventricle was distended with pitch-like black blood. The kidneys presented the same appearance as in the dog I first described.

*Therapeutical Applications.*—To a gentleman suffering with an acute attack of gout I administered one-forty-fifth of a grain of colchicina, three different times, at intervals of four hours. It produced no effect upon the bowels, but the urine was largely increased in quantity, and contained a very large amount of urate of ammonia and mucus. I could not perceive that it produced much change in the pulse. The dose was now increased to one-thirtieth grain, which I was obliged entirely to suspend after the third dose. The pulse fell in frequency twenty-eight beats, the urine continued to flow very freely, and still contained the same large amount of urate of ammonia and mucus; the bowels were opened several times, the discharges were of an ochre color, very frothy, and had a strong urinous smell; there was some tenesmus, and an inordinate amount of flatus, which rather amused him at first, but eventually became quite painful. As I remained some time with my patient, and saw no cause for fearing too severe an action, I gave nothing but large quantities of mild diluents. I had no occasion to repeat the medicine, as it completely arrested the paroxysm. The urine that was passed before the administration of colchicina was small in quantity, of very dark color, deposited uric acid in large quantity on cooling, and was of sp. gr. 1·021. That passed after the third dose of colchicina was large in quantity, of much lighter color, containing a very large quantity of urate of ammonia and mucus, and was of sp. gr. 1·030. That passed after the bowels had been very freely acted on was still large in quantity, and contained about the same quantity of urate of ammonia and mucus, and was of sp. gr. 1·025, thus making a difference in the amount of solid matters discharged of about twenty per cent. even in the same quantity of urine passed; but as the amount passed was certainly four or five times larger, the amount of effete matters carried off in this way must have been very great.

Another case of gout coming under my notice about the same time, I gave one-thirtieth grain of colchicina, and repeated it seven times, at intervals of from four to six hours. It acted more quickly on the bowels than in the previously mentioned case, producing the same ochre-colored, frothy, and urine-like smelling discharges, as before spoken of, and producing much flatus and some tenesmus. The increase in the quantity of urine passed was very marked; the sp. gr. increased from 1·018 to 1·024, and uric acid and mucus were deposited in large quantities. This is the only paroxysm that this gentleman has had.

There is one other person to whom I have administered the colchicina. This person was suffering from a subacute, or rather chronic, attack of gout; he had gouty concretions of urate of soda, and enlargement of the joints. I gave him one forty-fifth grain three times a day, for ten days. It acted freely on the bowels three or four times daily, producing flatus to such an extent that he had to keep watch on himself when any one was near. The urine was increased in quantity and in specific gravity, and deposited large amounts of uric acid on cooling. He was very much benefited by the treatment. I find no other mention of the use of this agent in the treatment of disease, excepting by Dr. Guensberg, of Breslau. He has used it in many cases since 1853. Patients that had long suffered from gout, took, during the painful paroxysms of the swellings of the joints, one-sixtieth of a grain (of Geiger's) three times daily. In every case the remedy acted as an intense excitant of the intestinal secretion, even in such patients who had always before suffered from constipation. After three or four weeks' use of the colchicin, patients who before had suffered from an attack every two or three months, remained entirely free for a year or longer. But in acute articular rheumatism its employment did, contrary to his expectation, but little or no good.

*Modus Operandi.*—We have not a very large number of physiological experiments from which we may draw inferences as to the modus operandi of colchicina; but the few experiments that are given demonstrate its effects with greater accuracy than is usual with new remedies. We see by the physiological experiments on animals of Geiger, Albers, Hoppe, Aschoff, Bley, and Schroff, that colchicina uniformly acts as an irritant to the mucous membrane of the intestinal canal, producing frequent and copious alvine discharges; that given in the quantity of one grain or over to the smaller animals, it universally caused death, with pathological evidences of gastro-enteritis. We see, also, by the experiments of these gentlemen, that it enters the circulation and produces upon the blood the changes that a mere acrid poison does not necessarily produce. Aschoff and Bley have demonstrated its existence in the secretions. All of the experiments performed, those of my own included, demonstrate that, although it induces vomiting, the vomiting only takes place after a considerable time, that it is first absorbed and that the vomiting is but the consequence of the gastro-intestinal irritation. Although it has been common to call colchicum an acrid narcotic, we see that it possesses no narcotic properties, that it has no special action upon the brain or spinal marrow, and that a very large increase of the dose but little increases the intensity of the symptoms, and does not hasten death. We see by the physiological experiments performed by myself, and also by the therapeutic action in the cases I have reported, that in addition to the effects above mentioned, we have an increase at first in the amount of urine discharged; but in poisonous doses the urine is soon entirely suppressed, owing to inflammation of the kidneys. This is not mentioned as one of the actions of this medicine by the gentlemen whom I have just quoted; but in the experiments I performed it will be remembered that no animal to which I administered it passed any urine after the fourth hour, and that after death none was found in the bladder. Upon examination of all the animals that I experimented upon, pathological changes, which alone were sufficient to cause death, were found in the kidneys; in two of them the whole organ was inflamed, and the congestion extended to the infundibula and pelvis. In the dog to which I administered tannin in connexion with the colchicina, the kidney was less inflamed than in the other animals, but a smaller quantity of urine was passed by this animal, and the desire to urinate was less urgent. After death the Malpighian bodies and interlobular plexuses were found highly congested. This was sufficient to prevent the elimination of any urine, and it appeared to me that the astringency of the tannin had had the effect to retard the passage of as much as usual of the poison through the kidneys. It will be seen in the cases in which I record the therapeutic action of the remedy, that the quantity of urine was largely increased, and that the effect was produced even before its action on the bowels; that in addition to the increase in quantity, there was also a very great increase in sp. gr., and that the amount of urates and mucus was very large. Guensberg, who alone in addition to myself has tried the therapeutic effects of this remedy, has only noticed that it acted as an intense excitant of the intestinal secretion; but his were chronic cases, which he probably saw but once a day; but he found that it produced absorption of the swelled joints. Schroff, who administered it by way of experiment to a person in health, states that "the urine was like whey, with abundant white sediment." It will be noted, then, that we have given several instances wherein, administered in medicinal doses, colchicina increases both the quantity, specific gravity, and uric deposit of the urine. Let us turn again to the character of the fæces discharged; all state it to be large in quantity, mucoid, and frothy, and when it has been particulary examined, I have stated that it has a strong urinous smell. This effect is as marked with the administration of tincture of colchicum as with colchicina; and once, some years ago, I examined the fæces of a gouty person while under the influence of colchicum, and found them to containe a large amount of uric acid. It will be remembered that *Chelius*, of Heidelberg, many years ago, endeavored upon theoretical reasonings to explain that colchicum cured gout by eliminating uric acid from the blood, because he noticed that under the action of colchicum the amount of uric acid in the urine was much increased. This is disputed by those celebrated men Dr. Pereira and Dr. Graves, who not only deny that colchicum augments the excretion of uric acid, but state that it rather diminishes it when the remedy is given to its full effect. This, in my opinion, is one of the best evidences in proof of the theory of Chelius, for the gentlemen just named carry their observations only so far as to state that under the full effects of colchicum the amount of uric acid in the urine is decreased; here their observations cease; they make no examination of, or investigation into, the character, amount, and composition of the alvine discharges, nor have they examined the blood before and after the administration of colchicum. As I have just stated, I have in one instance proved that tincture of colchicum administered to a gouty person, to its full purgative effect, produced the elimination of a large quantity of uric acid in the fæces; that the urine before the purging contained more uric acid than it did after. In other instances where it was administered in small doses, not sufficient to produce purging, the uric acid in the urine was greatly and persistently increased. In the cases which I have reported of the therapeutic action of colchicina, we find the quantity of urine increased, as well as the specific gravity, and that the urates were in great abundance. This occurred from the time of the administration of the dose until free purging was produced; then the specific gravity was less, and the quantity discharged less, but both were more than before the administration of the colchicina. Guensberg found colchicina reduced the gouty swellings, and for many years colchicum has been used to reduce the deposit of urate of soda, occurring about the joints. It would seem, then, to me, viewing the various effects we find produced by colchicina, that its *modus oper-*

*andi* consists in its removal from the system of a large amount of urates. Chelius stated this to be its effects by noticing the augmentation of uric acid in the urine only; I think I have demonstrated his observations to be correct, not only in the amount of urates, but in the increase of the specific gravity also, and also by its presence in the alvine discharges. But the excellent work of Dr. Garrod fully explains these facts. In poisonous doses it first stimulates the kidneys, then the intestines; and destroys life at last, not only from the inflammation it produces in these organs, but by its preventing any secretion of urine, and by its acrid, poisonous properties upon the blood. Could the kidneys continue their functions, it would all be eliminated, and the system would recover from the poison; but, like most acrid poisons, it inflames and paralyses the kidneys, and is thence retained in the system, changing the character of the blood. I need hardly discuss the question of its absorption. I have so frequently during the session given you demonstrable proofs, by physiological experiments, that this class of remedies is absorbed into the circulation before they produce their peculiar effects upon the system, that repetition here I deem unnecessary. Being absorbed into the system, its action is catalytic, producing some peculiar change in the character of the circulating fluid, stimulating certain of the excretory glands, and passing out of the system after it has produced its peculiar effects. Its primary effects are upon the blood, for we find, when given in too small doses to act upon the bowels, that it always stimulates the kidneys, and increases the amount of excreted metamorphosed tissue. That its action on the blood is of that peculiar character to cause a rapid elimination of this product, is proved by the increase of the urates in the urine, and by their presence in large quantities in the fæces. Its action on the bowels, then, though always hitherto spoken of as its primary action, I deem but secondary to that upon the kidneys; and when the kidneys are unable to eliminate either it, or the changed materials that it produces, the blood becomes so altered as to be unable to become arterialized, and is found in the heart and arteries after death black and pitch-like.

*Uses.*—From the physiological effects of colchicina we may ask, What are its uses? We have seen from several cases that it has given speedy relief in gout, and from the known effect of colchicum for many ages in that disease we have empirical as well as rational proof of its value. Colchicina has never been used in inflammatory rheumatism, but the testimony of thoughtful men is that colchicum is of no service whatever in that disease. From its physiological action we have every right to draw deductions that it will be found of great service in those diseases where uric acid and the urates are in abnormal quantities, and require to be removed from the system.

When speaking of the action of colchicum I told you that objections were raised by some against the use of it in gout, because it seemed to lose its effects in subsequent attacks. Is not this rather the nature of the disease than the want of proper action of the remedy? A first paroxysm of gout is frequently easily controlled in a short time, and by a mild remedy, but each successive paroxysm fixes the diathesis more firmly on the system, until after a time no remedy will cure or cut short the duration of an attack, it only palliates the pain. A certain length of time is required, and a certain amount of abstinence necessary to enable the medicine even to relieve the symptoms; the gout then disappears for a time, and returns again at its regular period. Even in these instances colchicum greatly relieves the severity of the pain, and is necessary before a cure is effected. Another error is frequently committed:—Colchicum, and it alone, without regimen or diet, is depended on, and as it gives relief nothing is administered afterwards to correct the still existing depraved condition; whereas, had proper after treatment been resorted to, the patient would not be left in a condition to find fault with the injurious action of any medicine. One thing is certain, a majority of the cases of gout we meet with are quickly cured by the action of colchicum, and in many other cases it affords great relief from the pain, and is frequently the only medicine capable of giving relief. It is as near a specific in gout as an other medicine in other disorders; but it will be recollected that there are no specifics. Guestenberg demonstrated that colchicina afforded great relief to old and chronic cases.

*Antidotes.*—It has generally been supposed that tannic acid was an antidote to the poisonous effects of colchicum. Acting upon this view, Aschoff administered 15 grains of tannin to a dog, to which he had previously given one grain of colchicina; it had no antidotal effects. It will be remembered that I administered 20 grains of tannin in combination with 2 grains of colchicina, and afterwards gave 30 grains more of tannin, and that it had no effect in preventing the action of the poison, or prolonging the life of the animal. From the rapid manner in which colchicina was absorbed by animal charcoal, Carter recommends it as an antidote, and if it could be administered immediately I have no doubt that it would be perfectly protective until means could be adopted to remove the whole from the stomach; but unless administered immediately it would be of no effect—because the absorption of the poison is rapid, and it would in no way counteract its action when once absorbed. Magnesia also has been recommended; but magnesia is very frequently given in large doses with tincture of colchicum, and yet the colchicum produces its peculiar effects. All that can be done is to counteract the effects of the poison, and this I conceive will be most successfully accomplished by full doses of opium and stimulents, with free diluents.

*Doses.*—Of the article made by Oberlin, and by myself, about one-twentieth grain should be the maximum dose. I found one-forty-fifth to one-thirtieth to be safe if not too frequently repeated. In these doses it produced promptly its characteristic effects, and had the advantage over any of the crude preparations that it was definite, and did not deteriorate on keeping. It is always difficult to get a good preparation of colchicum, and hard to keep it good. This, when once prepared, does not change, and is definite in its action.

RESINA PODOPHYLLI (PODOPHYLLIN).

*Physiological Effects.*—I am not aware that any physiological experiments that have been published have been performed on animals, either with the podophyllum root or with the resin. I have performed some few experiments on animals, but mostly to ascertain the purgative effects of the resin. With the fresh root I have tried no experiments either on man or animals, but from the descriptions found in the books, and from the relation of some few cases to me, it seems to produce great irritation of the intestinal canal, gripings, prostrating emesis, and catharsis, an irritable and frequent pulse, and profuse salivation. These irritant effects are produced by a volatile principle existing in the green plant and root, which is mostly dissipated on drying. The effect of the green root or plant, or the fresh decoction of them, upon the mouth and salivary glands, resembles in a mild degree that of the Arum triphyllum, and the profuse salivation produced is principally the effect of the local stimulation, for salivation is but very slightly induced by the dried root or resin, unless it is given to its emetic effect; then it acts as emetics in general, and freely increases the secretion of saliva. It has been so frequently asserted that podophyllin produces salivation that I have taken much pains to ascertain its action in this respect, and I found, when given in pills or capsules in small and frequently repeated doses, or in one large dose, that it has no persistent sialagogue action, and no effect like mercury, producing soreness of the gums, fœtor of the breath, and profuse and continued secretion of saliva. As I before stated, when given to its nauseant or emetic effect, it always induces a free secretion of saliva, but as its emetic action passes off so does its sialagogue action also. But if the resin is given in powder, so that it produces a local stimulation upon the glands, I have seen abundant secretion of saliva for one or two hours. In this way it is merely a topical irritant, not a true sialagogue. There are no means of ascertaining whether the resin when passed into the stomach in capsules can be detected in the saliva, but that it exists in the saliva, when administered by the mouth in powder there can be little doubt, for the resin is soluble in the saliva.

Of the commercial podophyllin (of Messrs. Keith's manufacture) I have given two grains to a dog; in eight hours it produced three free alvine evacuations. The same dose was repeated the next day, and it acted on the bowels in three hours, and during the day caused more than a dozen evacuations. To the same dog I administered, by hypodermic injection, under the skin of the leg, one grain of podophyllin dissolved in liquor potassæ. It produced great local irritation, free purging in two hours and twenty minutes, evident colicky pains, and much tenesmus, with retching, but no vomiting.

To a man suffering with constipation of the bowels I have sprinkled two grains of the resin in fine powder over a large indolent ulcer. It caused great pain in the ulcer, free catharsis in six hours, with nausea and severe griping pain. Within twenty-four hours it acted on the bowels seven times. The appearance of the ulcer was improved by the application.

Podophyllin, when administered to a person in health, is an efficient and certain cathartic; slow in its operation if administered in proper medicinal doses, but if administered in large doses quick and violent in its action, causing nausea, vomiting, and repeated and painful purging of mucous and billious matters. When taken in powder in moderate doses it is not very disagreeable when first put into the mouth, but as soon as the saliva dissolves a portion of it becomes disagreeably bitter and nauseous, and the sensation it leaves in the mouth and fauces is quite unpleasant; when taken in this way there is a free secretion of saliva for some time. When I have taken the powder finely rubbed up with sugar in this way there is no sensation experienced in the stomach for an hour or more, excepting the first sensation of nausea from the disagreeable taste. In about an hour, if it has been taken fasting, there is an uneasy feeling in the stomach, accompanied with nausea and free salivation. This lasts for about an hour, and it feels as though a large secretion of gastric fluid was being poured out, and the stomach feels as if in a state of commotion. Soon the influence is felt in the small intestines, and unmistakable sensations of the secretion of the bile are experienced. In this stage of operation it produces on me exactly the same sensations as I experience from a full dose of calomel. The influence continues to be felt through the whole length of the intestines, producing active peristaltic motions, and the sensation as though acrid bile was freely passing. In about five hours one grain will purge me quite freely, and this is followed within two hours by two or three free bilious evacuations, producing upon me the same sensations and the same bilious-appearing alvine evacuations that I experienced from the same proportionate dose of calomel. In this dose it does not gripe nor produce much tenesmus, but during the whole time of its passage through the intestines there is an unmistakable sensation of a dose of medicine producing a chologogue effect within. If the same dose (one grain) is taken immediately after eating, and protected in any way so that it does not touch the mouth, no effects whatever are felt from it for two or three hours; then the effects in the intestines above described are experienced in a very modified degree, and the result will be one copious pultaceous evacuation. The after effects in both instances are an increase in appetite, and a feeling of better health. Most persons will require a rather larger dose of the commercial article than this, and many can take three grains.

*Therapeutical Effects.*—Podophyllin was first and most largely used by the "Eclectics," and many of them have written intelligently upon its therapeutic applications. By the Eclectics it has been called Vegetable Mercury, and its use has been recommended in all diseases in which mercury has been found to be of service. To a certain extent, and in some of its effects, it certainly does much resemble that drug.

Its greatest use is in that class of diseases usually called bilious disorders; that is, in those disorders where the whole digestive organs are deranged. In these disorders a dose of one, two, or three grains of the commercial podophyllin will be found to excite the secretion of all the abdominal organs, acting as an efficient purgative by this increased secretion. The largest number of patients whom we are called upon to treat are suffering more or less from these disorders, and it has undoubtedly been too much the case to give some mercurial for their relief. In these disorders podophyllin, combined in the manner we shall hereafter describe, is fully as efficient to cause a free secretion from the intestinal mucous membrane, and from the liver and pancreas, as any of the preparations of mercury, and it is infinitely safer. There is a very grave accusation made against our Militia Army Surgeons for using too much blue pill and calomel in these disorders, and although the accusation is an unjust one against the majority of the surgeons there are undoubtedly some against whom it is too true. Our soldiers, who are so much exposed, should not use mercurials if it can be possibly avoided, and this article will, if properly given in these disorders, have a more beneficial effect, and will produce none of the evil consequences of mercury. There is but one drawback to its use, that is the inability of the patient being upon duty for ten or twenty hours after taking it owing to the nausea and tormina it produces if given in a full dose. In some of the forms of hepatitis it is of great value, and causes a full secretion of bile, but as this is not the only indication in the acute form of the disease, it cannot be relied upon to check the inflammation. In chronic hepatitis I have found it of very great service, acting better than any other remedy I am acquainted with, as it relieves the portal circulation by its action on the secernents. In this disorder it is not necessary, in fact it is frequently injurious, to give it in large and powerfully cathartic doses; I have found it better to give it in small and frequently repeated doses upon an empty stomach, sometimes combined with veratrum or hydrocyanic acid, at other times with strychnia or capsicum. The amount of bile and intestinal mucous secretion carried off by treatment of this description is sometimes enormous.

There are few diseases in which it is of more service than habitual constipation. In this disease small doses taken with the meals (frequently in combination with strychnia and capsicum) will in the majority of instances relieve the disorder within two weeks. It needs but proper graduation to give it in just the proper proportion.

From its thorough action upon the whole intestinal mucous surface, and upon the large glands, it is one of the best eliminants in infantile convulsions. For the same reason it advantageously follows the use of anthelmintics.

But as from the experiments I have made with it I will endeavour to give its mode of action, I would rather leave its application in diseases to your own judgment. If you know its physiological and therapeutic action, you can apply it intelligently in the treatment of diseases.

*Modus Operandi.*—We find that podophyllin consists of two resins, one soluble in ether, the other in alcohol, the resin which is soluble in ether being, in most instances, the most active. Both of these resins are soluble in solutions of a caustic alkali, and when so given act rather more quickly as a purgative than when given in combination with an alkali. It will be remembered, that when applied to an ulcerated surface it acted equally well upon the bowels as when given by the mouth, and that it also acted upon the bowels when injected under the skin of a dog. In my lecture on iodine I proved to you that the various secretions of the body had the power of decomposing, rendering colorless, and absorbing the insoluble iodide of amidine; the same is the fact with this resinous substance, which, although perfectly insoluble in water and saline solutions, becomes soluble in the saliva, gastric fluid, pancreatic and biliary secretions. It is absorbed into the blood, then, whether given in an insoluble powder, or held in solution by an alkali. So far as my experience goes, it acts with less irritation when given in solution in an alkali than when given uncombined with an alkali in either pill or powder; because in this way it is more quickly absorbed, flows freely over a larger surface, and thus causes less irritation of the mucous membrane. Most of the resinous cathartics act as drastic purgatives, and by their irritating action on the intestinal canal indirectly excite the liver, and stimulate it into activity, thus acting as cholagogues. The same result is produced in a very mild degree even in the process of digestion. But that this is not due merely to the irritation of the orifice of the hepatic duct, is proved by the dissimilarity of the operation by different materials that stimulate the duodenum in an equal manner. Again, we find that each particular medicine has its own peculiar operation, whether introduced directly into the circulation of the blood, or administered by the mouth. And we find with this agent that in whatever way administered it passes into the blood, is absorbed and carried through the system, producing its own peculiar action of exciting into activity the glandular system, and that by the augmented secretion of these glands it passes out of the blood, and removes from the system the effete matters secreted; thus doing good by removing not only from the bowels but from the glands the irritating materies morbi, no longer needed in the system, in this manner purging the blood. It is claimed for this medicine that it has an action on the liver similar to mercury, and owing to this asserted similarity it has been called vegetable mercury. How are we to arrive at the facts in this case? I am not aware that any chemical tests could be applied to ascertain the presence of so small an amount of this agent in the liver, for it will be remembered that half a grain of the pure ethereal resin is a full dose. We cannot then, as Buckheim did with mercury, actually detect it in the liver of dogs to which it was given. But we can (and have by many careful experiments) ascertain that bile exists in very large quantities in the alvine discharges of men and animals to whom the resin has been given; and reasoning from good analogy, we can assert that it has an active agency upon the liver, because diseases of the liver and bilious derangements are cured by its operation.

Again, it is claimed for this medicine that it has a powerful *alterative* action. Using this word in its fullest sense, we cannot but acknowledge that it produces the effects claimed for it, for as the term is used it signifies medicines which *alter* for the better the state of the system. From the action that the resin exerts upon the blood we have seen that it stimulates the function and increases the secretion of various glands, in this way *altering* the composition of the blood itself, and becoming a blood medicine by the change it produces in that fluid by its true eliminative action.

It is asserted by one practitioner, that he has cured one hundred and twenty cases of syphilis with podophyllin, and that it acts as well as mercury, without any of its injurious effects. It is undoubtedly obvious to many observers that there is a great decrease in the cases of syphilis of the true Hunterian character, and that a great majority that now exist are readily cured without the use of mercury by good local and general treatment. Of such as these were no doubt the cases here spoken of. Let those cavil who will, but it is an indisputable fact that syphilis is of a milder character here than it was twenty-five years ago; and the great majority of cases can be cured without any mercury, and podophyllin in mild cases of soft chancre would be better than most other remedies.

Of its sialagogue action we have spoken in another place.

It is to be regreted that so few physiological and chemical experiments have been performed with this medicine, for without them we are to a great extent ignorant of its true modus operandi. I have before promised you that, with Dr. Elsberg's assistance, I will at our earliest convenience give to the public a work on New Remedies and their Therapeutical Application. Before that work is published I will endeavor to institute a series of experiments, so that we may be able to give a more full and ample account than I can now do, and with this explanation you will excuse me for leaving unsaid many things regarding it that you would otherwise have expected of me.

*Doses, and Modes of Administration.*—As the podophyllin made by different manufacturers differs in its composition, the amount required for a dose will vary according to the sample that is taken. Again, it will vary in its action; for when the pure resins is taken alone it acts more quickly, and produces more pain than when given in combination with some carminative or sedative. Of the samples that are in the market the full purgative dose for an adult will vary from one to three grains. The amount necessary to be taken will vary of course with the effects required to be produced. If an ordinary dose of "bilious medicine" is required for an adult man, a very good pill will be:—Podophyllin, two grains; capsicum, two grains; both finely powdered and well rubbed together, and made into a mass with a small amount of honey. This pill may be taken at bedtime, and will generally operate in the morning, without causing much uneasiness. If a dose is required for a delicate female, a pill may be made in the following manner:—Pedophyllin, dried carbonate of soda, each one grain; extract of hyoscyamus, two grains. This will be moist enough to work into a pill, and may be taken at bedtime. It is difficult to get children to swallow pills. I therefore usually prepare a syrup in this manner:—Podophyllin, four grains; liquor potassæ, sixteen minims; syrup of ginger, one fluid ounce. The podophyllin, in fine powder, is rubbed in a warm porcelain mortar with liquor potassæ, and as saponification takes place the syrup is gradually added. For a child from six to ten years old, the dose will be a teaspoonful.

